# An Update on HIV Pre-Exposure Prophylaxis (PrEP) Among Women

**DOI:** 10.3390/v18060636

**Published:** 2026-05-31

**Authors:** Tamara Barnett, Daniel Cloutier, Rafique Van Uum, Parsa Ebrahimpoor Mashhadi, Agustina Crespi, Hadeeka Tahir, Sajeela Rana, Carlee Giffen, Roya Haghiri-Vijeh, Mia J. Biondi

**Affiliations:** School of Nursing, York University, 301A 4700 Keele Street, Toronto, ON M3J 1P3, Canada; tamarab@yorku.ca (T.B.);

**Keywords:** HIV, pre-exposure prophylaxis (PrEP), prevention, medication, access, women

## Abstract

Globally, cis and trans women face increasing rates of HIV, yet the uptake of existing HIV prevention medications often fails to meet their specific needs. This review examines HIV pre-exposure prophylaxis (PrEP) use among cis and trans women, including adolescent girls and young women; newcomers and migrants; sex workers; women who use drugs; and women who have been incarcerated, acknowledging intersectionality exists between these groups. A review of peer-reviewed published literature was conducted, and findings specifically on oral PrEP were synthesized. This review highlights several key themes shaping women’s engagement with PrEP, including barriers to initiation and discontinuation; public health messaging and promotion; the role of women’s networks; intimate partner violence; interpersonal trust in relationships; and “seasons of risk,” where temporary reductions in perceived risk may lead to discontinuation. Additional themes include preferred access points for PrEP, regional differences, and clinical implications for practice. Peer support and peer navigators emerge as important mechanisms for creating safe spaces that enhance trust and sustained PrEP use. Improving PrEP uptake and persistence among women requires a multifaceted, women-centred approach that addresses clinical, social, and structural barriers. Context-specific implementation remains critical to addressing diverse lived realities and strengthening HIV prevention outcomes globally.

## 1. Introduction

An estimated 40.8 million individuals worldwide are living with HIV, with women and girls representing 45% of all new infections [1]. Globally, cis and trans women face increasing rates of HIV, yet the uptake of existing HIV prevention medications often fail to meet their specific needs [2]. Indeed, HIV prevention strategies remain limited among women, particularly pre-exposure prophylaxis (PrEP), which is a well-studied oral combination therapy to effectively prevent HIV [3]. At present, PrEP options for cis and trans women include daily oral tablets, monthly intravaginal rings for cis women, and emerging long-acting injectables that are currently under study and approved in select regions [4,5]. While multiple HIV prevention medications exist, including post-exposure oral medications, this review specifically focuses on oral PrEP.

Large-scale systematic review and meta-analysis showed oral PrEP efficacy and effectiveness across all populations at risk for HIV [6] and high levels of adherence have been observed, particularly among men and serodiscordant heterosexual couples [7,8]. However, subsequent studies of oral PrEP among women have shown inconsistent effectiveness, with uptake and adherence emerging as a critical determinant of PrEP efficacy in this population [9,10]. A 2024 scoping review on PrEP use and adherence among adolescent girls and young women (AGYW) in Eastern, Southern and Western Africa found that although PrEP is highly effective at over 90% when taken consistently, adherence and uptake remained low due to social stigma, low HIV risk perception, and structural barriers to access [11]. Furthermore, a systematic review of 44 studies (34 studies from Eastern and Western European regions), including 4699 women, identified common barriers to PrEP use, including low awareness, low perception of HIV risk, stigma associated with HIV and HIV prevention, and concerns about medication interactions [12].

PrEP-to-need ratio (PnR), defined as the number of PrEP users per new HIV diagnosis, is a metric used in public health to reflect the portion of women using PrEP relative to their HIV risk [13]. In Canada, the PnR was recently found to be 1.2 for females compared to 22.6 for males, signalling a profound unmet need for HIV prevention among cis and trans women [13]. In the United States, only 36% of individuals who could benefit from PrEP were prescribed it in 2022, with coverage among women at 15%, compared to men at 41%, highlighting persistent gender disparities in access and utilization [14]. Likewise, in a study of 49 countries supported by President’s Emergency Plan for AIDS Relief (PEFAR) in Africa, Asia, Latin America, the Caribbean, Eastern Europe, the PnR ratio for women rarely exceeds levels that would indicate sufficient coverage, showing substantial unmet HIV prevention needs [15,16]. Evidence suggests that many women who could benefit from PrEP remain unidentified or are not offered it [17]. Restrictive and inadequate policies disproportionately affect women in low and middle-income countries [18]. These policies compound existing structural barriers with system barriers, including misinformation, limited awareness, structural discrimination, provider stigma, high associated cost, varying legal rights of women globally, and poor healthcare access [18]. In parts of Asia and Africa, policies that criminalize at-risk populations further perpetuate stigma and create significant barriers to access [18].

The current literature consistently highlights women’s recurring concerns and challenges with oral PrEP adherence, underscoring the need for further review and a deeper understanding of factors influencing their experiences and engagement with PrEP [19,20]. A key gap in the literature is the lack of a formal concept analysis to fully define adherence related to prophylaxis use, highlighting the need to conceptualize and operationalize prophylaxis adherence within nursing and public health literature. This gap is important as existing concept analyses are grounded in adherence to HIV treatment rather than prophylaxis. Drawing on Walker and Avant’s concept analysis of adherence in the context of treatment, adherence is characterized by attributes such as following a treatment plan, communication with healthcare providers, and willingness to engage in treatment, with antecedents including multiple medications, lifestyle changes, illness perceptions, and long-term treatment needs, and outcomes such as treatment success, improved long-term management, and better clinical outcomes [21]. Across studies, women’s experiences with oral PrEP are influenced by recurring themes, including key barriers, reported concerns, as well as gaps in public health messaging and promotion [22,23]. Social networks and community norms influence and shape adherence, while intimate partner violence, interpersonal trust between women and their partners, can hinder uptake and persistence [23,24]. Additionally, women’s HIV risk is dynamic and shaped by ‘seasons of risk’ [25], while global literature highlights preferences for specific PrEP access points, as well as important regional variations and clinical implications. Accordingly, this review synthesizes findings from literature on PrEP use, adherence, persistence, barriers and facilitators, we examine HIV prevention among cis and trans women (TW), AGYW, newcomers and migrants, sex workers (SW), women who use drugs (WWUD), and women who have been incarcerated. The findings of this review are organized around several key themes that collectively shape women’s engagement with PrEP. The themes include barriers to PrEP use and discontinuation, public health messaging and promotion, the role of women’s networks, impacts of intimate partner violence, interpersonal trust with partners and ‘seasons of risk’ where a temporary pause in PrEP may lead to discontinuation. Additionally, the review addresses women’s preferred access points for PrEP, and summarizes regional findings.

## 2. Overview of Barriers to PrEP Engagement Among Women

Many barriers exist in the context of engagement to HIV prevention, including individual, social and relational factors, as well as barriers related to healthcare systems, providers, and broader structural conditions [18]. At the individual level, factors associated with low PrEP uptake among women include limited awareness, knowledge, and low perceived HIV risk [26] and concerns about PrEP medication interactions and side effects. Medication concerns include pill fatigue [27], pill size, taste [28], and the fear that PrEP medication may be mistaken for antiretroviral therapy used to treat HIV [29,30]. WWUD and SW in New York City, at the individual level, reported co-occurring health conditions such as medical conditions, psychiatric diagnoses, and substance use and the burden of attending multiple medical appointments as barriers to PrEP use [31]. In a survey with women involved in the criminal justice system in Connecticut reported their primary concerns regarding PrEP medication as side effects (70.4%) and fear of incomplete effectiveness (57.6%) [32].

At the social and relational level, women also face barriers to PrEP, including distrust in the healthcare system [33], mistrust of medical research due to historical exploitation [34], and distrust of the pharmaceutical industry [35]. These barriers are further compounded by prior negative experiences within the healthcare system [36]. Additional challenges include the lack of PrEP integration into routine women’s health services [29] and limited availability of PrEP providers, which leads to off-site referrals [37] and creates transportation challenges for women [38]. Self-perceived stigma of PrEP and HIV represents a social-level barrier that impacts individuals’ behaviour and may discourage women from initiating and adhering to PrEP. In a survey of 597 women at Planned Parenthood clinics in the United States, 37% believed they would not receive approval from their families, friends or sexual partners, as PrEP use might be viewed as a sign of promiscuity [38]. Social level factors, including PrEP stigma perpetuated by family members and peers, as well as frequent relocation of individuals between neighbourhoods, towns or regions, were found to be a key barrier for PrEP persistence among pregnant AGYW in Cape Town, South Africa [39]. In the United States, among Black women aged 13–24 in Chicago, the association between taking PrEP and being reminded of HIV was identified as a barrier to PrEP use at the individual level [40]. Similarly, in Malawi, 36% of adolescent girls aged 10–16 interested in PrEP reported individual-level concerns such as forgetting to take the medication (21%), as well as social-level concerns, including privacy (7%) and fear of parental discovery (3%) [41]. Furthermore, the need to hide PrEP from partners and family members was described as an additional ongoing social burden [42]. At the structural level, the cost of PrEP and/or financial hardship [39,40], competing financial priorities and fear of PrEP use disclosure through parental insurance [43], were found to be barriers.

Unstable social support, including romantic, social and parental relationships [39], including women’s caregiver responsibilities, may also act as a barrier to PrEP access, adherence and persistence [44], as many women are primary caregivers for children, partners, or other family members [45,46]. According to the United Nations, globally, women perform 76% of all unpaid caregiving and domestic tasks [45] limiting their autonomy, time, and the flexibility needed to attend medical appointments, complete required laboratory testing, or regularly refill prescriptions required for PrEP. These responsibilities may also make it difficult to prioritize their own health needs, leading women to delay or forgo seeking PrEP services [47]. A systematic review of 18 PrEP studies found that women reported their male partner’s influence as a key part of their decision to continue or discontinue PrEP [48], indicating the influence and importance of romantic partnerships in women’s decision-making when considering PrEP. Further studies suggest that supportive parental relationships are associated with greater adherence, where unsupportive relationships contribute to reduced adherence, an important barrier across the HIV prevention cascade [11,49]. Qualitative research with TW in Southern California reported a lack of provider competence in delivering gender-affirming care, such as limited knowledge of TW’s anatomy [36]. This dynamic created a breakdown in the provider-client relationship, as TW found themselves having to educate their providers about their own reproductive anatomy, while their providers appeared confused and uncertain [36]. Furthermore, structural barriers, such as housing instability, create living conditions where belongings are kept in public spaces, leading to PrEP being stolen while in shelters [36]. Access, adherence, and persistence to daily oral PrEP remain a common challenge for women, particularly in the context of unmet social determinants of health, including poverty, systemic racism, unstable housing, substance use, intimate partner violence (IPV), stigma, and fear, which collectively hinder women’s engagement with PrEP worldwide.

## 3. Discontinuation

Discontinuation related to PrEP side effects and long-term impacts is mentioned consistently in studies investigating PrEP adherence among women [31,48,50,51,52,53,54,55,56,57,58,59,60,61,62,63]. Although studies often reported side effects non-specifically, commonly cited issues leading to discontinuation of PrEP included nausea, vomiting, dizziness, and fatigue [58] and although rare, elevated serum creatinine and liver enzymes also impacted adherence, despite PrEP not being cleared through the liver [57]. Concern regarding drug interactions with PrEP and hormones or hormone replacement therapy emerged as a recurring concern across multiple studies spanning multiple regions in the United States, Thailand and China [51,52,56,60]. While not evidence-based, this misconception further contributes to reduced uptake of PrEP in cis and trans women. The concern around hormonal interactions is particularly prevalent among trans women who report prioritizing gender-affirming hormone therapy over HIV prevention medication [56]. Hormone interaction concerns may explain why PrEP adherence trends lower among trans women compared to cis women [48,60], with rates of 72% versus 86% reported in the same study [60]. Women have also expressed the need for discreet, unmarked packaging for HIV prevention medications to reduce concerns with HIV related stigma and to protect privacy [64,65]. Unmarked packaging could support the uptake of HIV prevention options in real-world settings [64,66].

Pregnancy was frequently cited as a reason for discontinuing PrEP, either due to being too preoccupied with pregnancy to continue [62] or fear of pregnancy-related side effects, and risk to the fetus [55,62,67]. Women also expressed worry and anxiety that taking PrEP could result in their partner discontinuing condom use, along with fear of conflict in the relationship [68]. In a qualitative study of AGYW aged 16–25 in South Africa, community stigma associated with unintended pregnancy, as well as fears of socioeconomic hardship and social isolation, were found to undermine motivation to continue on PrEP and attend regular follow-up appointments [18]. Together, these factors highlight the complexity of personal health concerns, maternal responsibilities, and relationship dynamics that can affect PrEP adherence, persistence and discontinuation during pregnancy.

## 4. Public Health Messaging and Promotion of PrEP

The promotion of PrEP as an HIV prevention medication for gay, bisexual, men who have sex with men (gbMSM) excludes women, leading to marginalization [27,69,70,71]. The lack of targeted social marketing highlights the need for higher-level interventions that reshape and expand public health promotion and messaging for PrEP, to better reflect the diverse identities, lived realities, and needs identified by women [49]. In the United Kingdom, research has shown that women are more likely to consider themselves as PrEP candidates if they are exposed to diverse, women-focused PrEP advertisements [72]. In South Africa, the 3Ps (Perception, Partner, Pills) for prevention study aimed to increase PrEP uptake and adherence among AGYW through social media marketing, focusing on power, protection and pleasure rather than fear and risk-based messaging [73]. Furthermore, SW in South Africa reported taking PrEP as an expression of self-love and personal agency [74], showcasing the importance of framing PrEP as a tool for autonomy and empowerment. While studies in South Africa demonstrate the value of empowerment-focused PrEP messaging, broader social marketing campaigns are needed to reach diverse populations of women.

In the United States, awareness of PrEP is generally lower among cis and trans women when compared with cis men [49,55]. A rapid review of 8 studies, primarily from the United States, with additional research from South Africa and Kenya, reported low PrEP awareness across Black cis women, TW, and women from other HIV-vulnerable communities in urban regions [67]. Young Black women aged 13–24 in Chicago reported television and radio (40%) as the most common source through which they had heard about PrEP, followed by healthcare professionals (32%), friends and/or family members (26%), and social media (26%) [40]. A study focused on 112 Black cis women in Chicago found low community awareness, stigma concerns, side effect fears, and worries around PrEP use during pregnancy as barriers, with minimal evidence supporting barriers related to cost or adherence [55]. Although a relatively small sample size, low community awareness, stigma, side effects and PrEP use during pregnancy could be addressed by tailored public health messaging and provider education. Larger studies, including samples of 1293 TW, 317 trans and cis women, and 65,324 trans and cis women from across the United States, emphasized the same concerns and need for PrEP education and awareness [10,60,75].

Notably, the common concerns, challenges, and confounding factors faced by subpopulations of women, for example, TW, cis women, women of colour, and criminalized populations like SW, and WWUD, are not identical or interchangeable, even with the same HIV risk factors present [31,55,56,58,59]. These findings highlight the need for PrEP outreach strategies to be adapted based on the specific needs of women across different demographic groups [49]. It is recommended that advertising and messaging related to women’s motivations for using PrEP, such as agency [76], empowerment [77,78], desire for condomless sex, protecting children and/or partner and future family planning [78,79] should be framed in ways that affirm women’s autonomy, reduce stigma, and position PrEP as an HIV prevention strategy for holistic sexual and reproductive health.

## 5. Harnessing the Power of Women’s Networks

Globally, PrEP programmes for women, including AGYW, newcomers and migrants, SW, WWUD, and women who have been incarcerated, benefit from leveraging women’s networks as a way of taking care of one another by providing mutual support [80]. In addition, community-led peer education has been cited as key to delivering trusted PrEP messaging and information [81,82]. For example, caregivers were identified as key allies in implementing PrEP for AGYW in Malawi, with 85.9% of caregivers expressing encouragement and offering decision-making guidance to support their AGYW taking PrEP [41]. A systematic review on the PrEP care continuum among SW and WWUD reported a strong preference for peer educators who provide community-based outreach, education and system navigation as preferred sources of PrEP information [83]. Women in the Southeastern United States agreed that a salon-based intervention to promote the uptake of PrEP would be acceptable based on trust in their hair stylists and social networks [84]. In Brazil, TW expressed a preference for PrEP program delivery using peer-based educators who could share their lived experiences, build trust, and help women feel understood, affirmed, empowered, and supported [85]. These peer educators were viewed as more trusted sources of information than traditional healthcare providers, with many TW expressing a strong preference through qualitative focus groups to avoid medical settings due to anticipated and enacted stigma [85]. In Vietnam, the Healthy Markets project assisted the Vietnam Network of Transgender People, TW influencers, and key-population-led (KP-led) private clinics to launch a dedicated campaign known as “Be Me, Be Happy” [61]. This campaign focused on creating an inclusive online and offline community for information and support on healthcare, well-being, and PrEP among TW. The program provided an online space where TW, considering PrEP, could ask questions to peer PrEP experts and healthcare providers [61]. PrEP adherence after three months was maintained, and the project was considered a success due to peer support [61]. This reflects a growing interest in virtual, peer-supported models for PrEP information and support [27,86,87]. Community awareness and education delivered by peers of Black and Latina TW in Los Angeles through culturally appropriate messaging/advertisements has the potential to improve PrEP knowledge, dispel myths, and reduce stigma [53]. This approach also provides motivation for use and supports adherence and persistence by addressing common concerns around side effects and gender affirming care [56]. Consistent with this approach, the World Health Organization (WHO) recommends involving community members and peers in the design and delivery of PrEP service to minimize PrEP-related stigma [88].

## 6. The Impact of Intimate Partner Violence on PrEP Access

The WHO defines intimate partner violence (IPV) as behaviour that causes physical, sexual or mental health harm or suffering to women, including coercion and deprivation of liberty [89]. Violence against women is a human rights violation rooted in gender inequality, leading to profound suffering. IPV affects 26% of ever-married or partnered women aged 15 or older worldwide, with 682 million having experienced intimate partner violence at least once in their lifetime [90]. In the context of PrEP, IPV is defined as a pattern of abuse or coercive behaviour involving partner interference that influences HIV risk, women’s HIV prevention decisions, willingness to use PrEP, interruptions in PrEP use, and adherence [67,91]. A narrative review including cis and TW highlighted concerns that PrEP may inadvertently encourage women to remain in unhealthy relationships and continue to have sex with ‘risky partners’ [49]. The intermittent and cyclical nature of IPV may also influence women’s PrEP decision-making and HIV prevention use experiences [67], as IPV can limit agency while often remaining unrecognized as a risk factor for HIV acquisition [24]. A qualitative study in Rochester, New York, examined the feasibility and acceptability of PrEP among women experiencing IPV and found that HIV prevention was not a priority and underscored the need for universal IPV screening protocols, enhanced provider education [92], and PrEP guidelines that included women experiencing IPV as a key population for HIV prevention [93]. Given that, women may have limited autonomy in sexual decision-making and may be reluctant to disclose their experiences of IPV due to its sensitive nature. PrEP programmes must be designed to reach, support, and retain women experiencing IPV.

Furthermore, a 2020 study indicated limited research on women’s PrEP use in the context of IPV, highlighting the urgent need for targeted research, public health interventions, and policy measures to address HIV risk and the specific needs of women who experience and are affected by IPV [67]. The WHO estimates that 1 in 3 women has experienced physical and/or sexual violence [90], and PrEP programmes and providers represent an important entry point for women experiencing IPV. Providers can discuss the risks and benefits of PrEP, while providing trauma and violence-informed care, including offering safety assessments, counselling, and referrals to appropriate intimate partner violence prevention and support services [88]. Although new biomedical HIV prevention strategies, such as long-acting injectable PrEP, have the potential to advance the global HIV prevention response, research and public health programmes must also address the underlying social factors that continue to drive HIV risk for women experiencing IPV.

## 7. Interpersonal Trust Between Women and Their Partners

Understanding the relationship dynamic between trust in a partner and PrEP use could enhance PrEP programme retention among women, as discontinuation of PrEP is highly nuanced [94]. As mentioned, TW have lower adherence compared to cis women. However, adherence to PrEP was found to be lower and discontinuation higher among cis women compared with cis men [49]. The observed lower adherence rate demonstrates a critical knowledge gap regarding the significance of socioecological factors and how they may influence adherence outcomes. When trust in a partner is reported in studies related to PrEP adherence and usage, it presents in a multitude of ways, including trust in a newfound partner, mistrust of a current partner, or mistrust targeted toward women by their partners [48,63,91]. Trust has dynamic impacts on PrEP adherence and can alter HIV prevention behaviours. In a multi-study site conducted in Philadelphia, Birmingham, and Washington (United States), participants no longer saw the need for PrEP once they entered a mutually monogamous relationship [63]. Potential shifts in trust dynamics and experiences of IPV may increase or decrease PrEP adherence, accelerate erosion of trust, or lead to a partner’s HIV status disclosure. Although a small sample size, approximately 70% of women in a study of 145 women in the United States reported willingness to use PrEP, with the key reasons for use including lack of, or dishonest conversations with partners [91]. Adult TW, from the United States and China, reported their reasons for not being interested in using PrEP were the fear that their male sex partner would consider the use of PrEP as a symbol of distrust or infidelity [48]. Similarly, cis and trans women in the United States reported that taking PrEP would make their partner upset and suspicious [49]. In a qualitative study, AGYW in South Africa and Zimbabwe reported that male partners accuse them of having other sexual partners because of their PrEP use [95], highlighting how partner reaction can create conflict and barriers to PrEP use, adherence and persistence. Partner dynamics in a qualitative study with AGYW in Tanzania emphasized the need to examine further and deconstruct gender and cultural norms that often deny women self-efficacy to access and use PrEP and to better understand the dynamics between AGYW and their male partners [96]. Power struggles, trust, concerns about a partner’s infidelity, monogamy, and anticipated partner reactions may either encourage or discourage women’s intentions to use PrEP [11,25,97]. Trust in a partner is an important social factor that impacts women’s PrEP use, adherence and persistence globally.

## 8. “Seasons of Risk”: A Pause May Lead to Discontinuation

Women may choose to take PrEP not only based on objective risk factors but for personal ‘peace of mind’, as knowing that they are protected against HIV can reduce worry about potential HIV risk [98] and may reflect a ‘seasons of risk’ approach to prevention. The concept of ‘season of risk’ which may encourage or discourage women from using PrEP recognizes that a women may use PrEP only during perceived times of increased HIV risk such as during the dating period, the beginning of a relationship, navigating new partners with unknown HIV status, inconsistent condom use, when trying to conceive, and when experiencing low trust in a relationship (suspected infidelity) and may discontinue PrEP when the ‘season of risk’ has ended [25]. In Eswatini, a qualitative study focusing on PrEP discontinuation reported pregnancy as the ‘season of risk’, when the study participants felt they could not negotiate condom use, therefore needed PrEP to protect themselves and their child from HIV, and discontinued PrEP at birth until the culturally expected 10-week postpartum abstinence period ended, after which PrEP would need to be resumed [99]. Periods of increased HIV risk occur among female partners of migrant miners in Mozambique when miners return home for the Christmas holidays, representing a ‘season of risk’ requiring heightened HIV prevention [100]. Additionally, life events such as mental health challenges, diagnosis of a sexually transmitted infection, and changes in relationship dynamics can trigger heightened HIV concerns that may not persist once circumstances change [101]. Furthermore, discontinuation of oral PrEP is complex and may align with periods of low or absent HIV risk, suggesting that pausing or stopping PrEP may reflect deliberate, complex context-driven decisions rather than nonadherence [94].

## 9. Women’s Preferred Access Points for PrEP

When women are given the option to choose where they would prefer to access HIV prevention medications, many express a preference for community-based settings [65,75], which aligns with the WHO recommendation to offer PrEP in community-based venues to support timely and accessible care, including task-shifting to non-specialist healthcare providers and community health workers [2]. Women have distinct preferred access points for HIV prevention services, often favouring community-based or non-clinical settings such as community health centres, mobile vans, harm-reduction sites [102] with co-located care and trusted staff [103], peers and peer support [102,104,105], community outreach [101,106], and high-quality provider-client relationship [39] over traditional hospital or specialty sexual health or HIV clinics. Specifically, AGYW in Kenya, Tanzania, South Africa, and Zimbabwe preferred access points include youth-friendly community-based services [73,107,108], including mobile clinics/vans [108,109] near schools or villages, and showed preferences for choices including HIV self-testing during PrEP use over testing by a healthcare provider [109]. WWUD in the United States and Europe recommend offering PrEP at harm reduction services and needle/syringe programmes [82], while SW worldwide prefer peer-led community outreach [56,110]. Some pregnant people prefer accessing PrEP where they are accessing their prenatal care [111], as women in Louisiana, United States, have stated they feel more comfortable talking about PrEP with their obstetrician-gynecologist (OB/GYN) than with their primary care provider, highlighting the importance of trusted providers in facilitating PrEP conversations [112]. TW prefer to access PrEP where they access gender-affirming care [113]; however, these preferences can vary regionally based on local context and service availability. Studies show AGYW clearly prefer having HIV prevention offered in safe spaces free from stigma, violence, abuse and stress and within supportive spaces in community-based ‘girls-only’ meeting venues. These environments offered a mentor or peer from the same area, which helps reduce power imbalances by fostering peer-to-peer connection [114,115,116,117]. Preferred access points align with women’s desires for safe spaces that provide physical and emotional safety where women feel protected from judgement, harassment and stigma, with key features being privacy, confidentiality, user-friendly, accessible locations designed to protect women from community-level harms. Supportive spaces include peer-supported and responsive care models that offer privacy, convenience, opportunities to build social networks, and an opportunity to gain life skills, suggesting that integrating PrEP into such settings can better support women’s uptake and sustained use of HIV prevention [118].

## 10. Findings by Region

### 10.1. Central and South Asia

#### Ho Chi Minh City, Vietnam, India, Malaysia

In the Central and South Asia region, greater odds of PrEP awareness were associated with discussion of HIV prevention with healthcare providers, higher HIV knowledge, and discussion of PrEP among friends [78,119,120]. Economic strain was reported in multiple studies as a barrier to PrEP uptake and adherence, with lower odds of PrEP awareness associated with unemployment [78,119,120,121]. A study conducted in India identified facilitators to PrEP uptake, including long-acting PrEP and discreet pill-based packaging, because it offered control and confidentiality [78]. Across multiple studies, participants consistently expressed a preference for privacy, as stigma was a persistent, recurring concern [42,78,119,121]. In a feasibility study, two hundred (approximately 47%) of 427 SW named people seeing and/or questioning their PrEP use as a potential challenge to adherence [121]. In addition to privacy measures, concerns also included the burdens of PrEP use, described as cost, adherence to daily medication, and hiding PrEP from family members [42]. The rollout of PrEP outreach programmes for this region should address stigma, monitoring, and delivery preferences [78]. Although many factors related to adherence and uptake appeared to be similar, differences in willingness to use PrEP were evident. In Malaysia, a cross-sectional survey reported a negative association between willingness to use PrEP and Chinese-Malaysian ethnicity, lifetime injection drug use, and older age with PrEP uptake and adherence of TW [120]. Chinese Malaysian ethnicity and reasons behind the differences remain underdefined and signify the need for further research of ethnoreligious and sociocultural nuance [120]. Furthermore, the negative associations observed in other populations indicate a possible need for individually tailored PrEP programmes to reach specific demographics.

### 10.2. Eastern and South Asia

#### Chiang Mai, Thailand, Vietnam, Cambodia

In Eastern and Southeastern Asia, education on HIV risk and the need for focused PrEP promotion campaigns is highlighted. A study involving 1466 SW found that substantial efforts are still needed to promote PrEP education, due to major socioeconomic barriers and limited access to information [122]. Overall, poor knowledge and understanding of HIV risk and prevention are likely major barriers to PrEP implementation in China [122,123], with low awareness and willingness to use PrEP among SW being influenced by these gaps in HIV risk and understanding [122]. Education on HIV risk appears to be a recurring need across the region. Among 230 TW in Thailand, a lower level of education was associated with lower PrEP retention, while those at moderate to high HIV risk were most likely to drop out of the PrEP programme [124]. Although PrEP uptake was modest in this study, adherence declined over time, particularly among younger TW clients and those with lower education, highlighting the need for stronger retention strategies [124]. In a study of 405 women, 50.9% believed they were not at risk of HIV through sex work [123]. In a study of 1003 SW’s, those who had heard about PrEP prior to the study were more likely to be willing to use PrEP. Willingness to use PrEP was higher in TW over 24 years of age (70% vs. 62.9%, *p* = 0.02) [125]. Among 1003 women surveyed in Cambodia, 63.2% preferred a non-government organization (NGO) as the access point for PrEP due to participants’ negative association with government-organized healthcare [125]. Some participants engaged in SW reported being hesitant to engage in government programmes but were willing to use NGOs [125]. As PrEP programmes continue to improve, studies in this region speak to the importance of targeted promotion campaigns that raise awareness of PrEP among TWs and outreach to entertainment venues where TW dance, sing, and converse. Intervention research is also required to identify the most effective ways to reach TW and to ensure adherence to PrEP [126,127].

### 10.3. North America

#### United States of America

Studies with 1293 participants from this region, along with others, expressed the need for trans-specific PrEP guidelines, as the risk of HIV is often underestimated, contributing to low uptake and poor adherence [10,128]. Barriers discussed in the region included side effects, medication cost, burden of taking a daily medication, and the reaction of peers to taking HIV medication [10,38,128,129]. A study of 679 women found that motivation for PrEP adherence was driven in part by anxiety reduction, and participants expressed a desire for PrEP to be discussed as part of routine care [130]. Stigma was reported, including historical experiences related to racism, which created mistrust in institutions, including healthcare, which further complicated access and adherence to PrEP. Stigma surrounding HIV and PrEP impacted Black TW in Chicago, Illinois, USA, in their interactions with healthcare providers [131]. Internalized transphobia and racism were factors impacting the group of women when interacting with their providers. These experiences undermine the trust necessary for open discussion about PrEP and sexual health needs, leading to barriers in uptake and adherence. These systemic barriers were also mentioned in another study with Black and Latina women from Chicago, citing a gap in PrEP marketing toward women, the importance of trust in a provider, and the importance of peer engagement in PrEP usage [59,132]. A study conducted in Connecticut found that women attending Planned Parenthood clinics reported that using PrEP could lead peers to perceive them as promiscuous, and they anticipated disapproval from family and partners [38].

### 10.4. Latin America and the Caribbean

#### Brazil, Lima, Peru

The need for education regarding PrEP, decriminalization of SW, and outreach programmes to mitigate stigma and barriers to access was highlighted in the Latin American and Caribbean regions. Compared to TW with high HIV risk perception, those with low HIV risk perception reported more significant concern about the potential interaction between PrEP and hormones. Demonstrating the need for further education and outreach. Fear of stigma was reported among 17.8% of TW [133], while lower PrEP awareness was associated with unprotected receptive anal intercourse and newly diagnosed HIV infection [69]. Lower odds of PrEP willingness were associated with concerns about the long-term effects of PrEP and with difficulties in access to healthcare due to transphobia [69]. In the region, TW were reported to have a significant history of facing trans discrimination within the universal healthcare system [50,104,134,135], which has contributed to limited PrEP uptake and adherence [50,134,136].

Studies suggested a successful HIV prevention strategy for TW may consider country or region particularities, addressing social and financial barriers, trans-tailored interventions supporting PrEP education, engagement, and adherence [50,134,136]. Decriminalizing sex work is urgently needed to advance human rights and strengthen HIV prevention efforts in Jamaica, which may also reduce stigma and promote greater PrEP uptake [137]. Those engaged in sex work who experience multiple vulnerabilities from stigma and criminalization are likely to face the most significant barriers to PrEP within this region [135,138]. Participants in a study conducted in Brazil expressed a preference for PrEP programmes and interventions that emphasized a gender-affirmative approach, promoted autonomy, and aimed to reduce stigma and discrimination in public health services [85]. The consequences of discrimination have created barriers for diverse populations of women, leading research to note that ‘one-size-fits-all’ strategies are unlikely to succeed [50,104].

### 10.5. Sub-Saharan Africa

#### South Africa, Kenya, Uganda, Tanzania, Botswana

Facilitators for PrEP use in Africa, where empowerment and autonomy with PrEP were seen to independently prevent HIV without relying on partners’ actions and condom negotiation and allowing for control of their sexual health [74]. Women viewed PrEP as an expression of self-love that aligned with broader life goals such as hope, future aspirations, longevity and parenting, while SW viewed PrEP as a way of supporting both health and income by maintaining their bodies as a source of livelihood [30,74]. Community norms strongly influence PrEP use, with support from family, friends, peers, and partners facilitating uptake and adherence, while community stigma leads to hiding pills and being perceived as living with HIV, and concerns with being viewed as promiscuous or unfaithful [139,140]. A study of 50 women in Bondo, Kenya, and South Africa found that the colour of PrEP pills serves as a facilitator while being associated with good adherence [141]. Women valued options in PrEP modalities (oral pills, intravaginal rings, injectable PrEP), and non-judgmental and trusted healthcare delivery increased confidence in PrEP [142]. Pregnant and postpartum women valued clinic visits that discussed PrEP and supported community outreach [140]. Maternal motivation, family support, pill bundling (take PrEP with prenatal medication), and tailored support during pregnancy and postpartum were found to be critical to sustain PrEP persistence in AGYW in Kenya [143].

### 10.6. Multiple Regions

#### Argentina, Malaysia, Brazil, Mexico, Peru, Philippines, China, South Africa, Kenya, United States of America, Vietnam

A global scoping review [144] reported that increased awareness of PrEP was associated with older age, higher education, and higher income levels. Globally, a high willingness (80%) to use PrEP was found, yet uptake and adherence were low (35.4%) [144]. PrEP awareness was more common among those who experienced hardship, such as a history of violence, involvement with carceral settings, sex work, and those who had STIs, and/or substance use challenges [144]. This study touched on multiple regions, including the United States, Brazil, Vietnam, South Africa, the Philippines, Thailand, Argentina, Malaysia, and China. In the Philippines, non-Catholic religious affiliation was associated with greater odds of PrEP awareness. In China, PrEP awareness was positively associated with lower sexual risk-taking behaviours, such as avoiding alcohol before or during sex. For this population, PrEP awareness was viewed as part of broader safe sex practices. However, Chinese ethnicity was associated with a lower likelihood for PrEP adoption [144]. At the interpersonal and structural levels, peer interactions, including lesbian, gay, bisexual, and trans (LGBT) specific social participation, were associated with greater odds of awareness among TW in the Philippines [144]. While having health insurance was associated with greater odds of PrEP awareness [144]. Furthermore, TW who accepted HIV services such as HIV tests, engaged in discussions with their healthcare providers and had high HIV knowledge had greater odds of PrEP awareness [144]. Early lessons from the accumulation of studies include the importance of marketing and education; integrating PrEP into youth-friendly clinics; strategies to improve adherence are essential; strategies to help AGYW; and assessing their needs and risks are important [19,52,144,145].

## 11. Clinical Implications

Understanding the multifaceted barriers and facilitators of PrEP use among women across diverse settings and contexts has important clinical implications for designing women-centred interventions, improving PrEP uptake and supporting sustained HIV prevention. Despite low PrEP awareness among women, studies show that women once informed of PrEP may be interested in taking it [146,147]. This suggests that proactive sexual health conversations for women must extend beyond contraception, pregnancy, gender-affirming care, and sexually transmitted infections (STIs) to incorporate routine PrEP counselling as part of a holistic approach to sexual health and HIV risk reduction [79,148]. However, studies have shown that clinicians often have low comfort, clinical knowledge gaps, and hesitancy in initiating PrEP discussions with women [149]. Lack of clinician knowledge, education, training, and clinical support has been identified as a key barrier to PrEP prescribing and uptake among women [150]. Women’s clinical considerations underscore the need for targeted provider-level training and gender-sensitive interventions to improve awareness and accessibility of PrEP.

**Figure 1:**
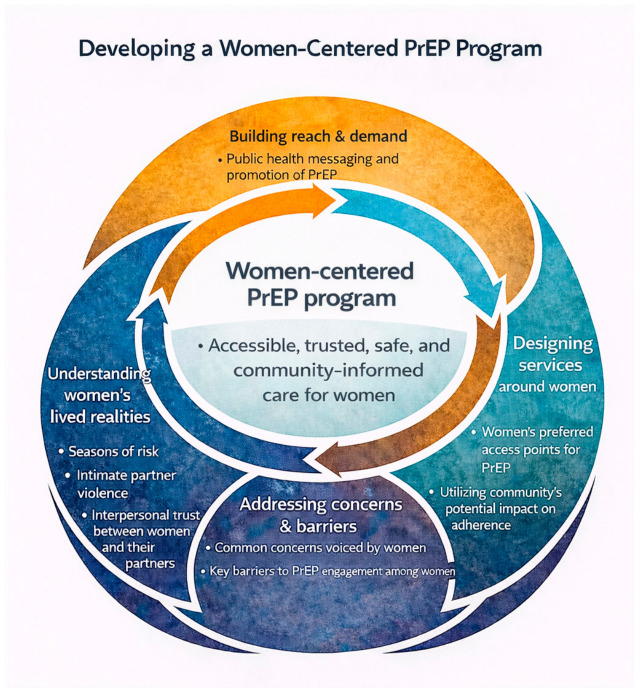
Conceptual framework for women-centred PrEP delivery and engagement.

Going forward, targeted PrEP programmes will need to incorporate clinical consideration while also supporting further research into emerging and context-specific population nuances. For example, clinical studies of PrEP in TW are needed to collect more detailed behaviour and anatomy-specific data, including participant engagement in receptive neovaginal sex following genital reconstructive surgery. Current evidence remains understudied concerning key biological questions regarding HIV susceptibility in the neovagina [151,152]. Addressing these sensitive topics is crucial to meeting the needs of TW and may help reduce stigma and build trust within this marginalized population [10,52,85]. This is also necessary to support providers in delivering evidence-informed care and to be better prepared in their client interactions. Ultimately, expanding inclusive research and tailoring interventions to diverse populations will be essential to improve PrEP uptake and reduce HIV transmission. Figure 1 demonstrates a woman-centred PrEP delivery and engagement.

Integration of PrEP into specialized HIV clinics has been shown to create barriers by reinforcing stigma and fear of being perceived as seeking HIV treatment, whereas integration of PrEP into broader health services has been shown to facilitate uptake [18]. Normalizing PrEP in settings that do not primarily serve people living with HIV or gbMSM by integrating PrEP into services that reach and support women [153] can help normalize and increase PrEP use in communities, as community acceptance is a facilitator of PrEP use among women [41,154]. PrEP implementation strategies suggest targeting clinicians’ attitudes, knowledge and competencies on PrEP care specifically for women [150,155] while building capacity to provide trauma and violence-informed care. Addressing clinicians’ biases and knowledge gaps, improving clinicians’ communication skills, and using inclusive language can reduce stigma and improve patient interactions. Normalizing routine discussions of PrEP with women regardless of perceived risk has been identified as a strategy to improve patient-centred access and reduce stigma while creating opportunities for shared decision-making between provider and client [156]. Furthermore, simplified, decentralized, and de-medicalized PrEP services may increase acceptability and accessibility for women while supporting PrEP uptake, adherence, and persistence [157,158]. The WHO Implementation Guidance for Simplified and Differentiated Service Delivery of PrEP [159] highlights that offering PrEP through flexible delivery models such as community pick-up sites, pharmacies, and home-based distribution can further improve the acceptability and accessibility of PrEP. Simplified PrEP delivery through pharmacies, community vending machines, home delivery [160], and over-the-counter PrEP are emerging discussions reflecting a demand for de-medicalized PrEP access, and these approaches may overcome challenges such as travel distance, transportation challenges, and the time required to attend clinics. Integrating PrEP into settings women are already accessing, such as community-based services, primary care, and harm reduction programmes, can further enhance both uptake and persistence. Additionally, providing supportive services that address broader social and structural challenges, such as unstable housing or assistance for women experiencing IPV, is critical to enabling engagement with PrEP and overall sexual health care. Peer support, including peer navigators, may also play a critical role in creating safe spaces that foster trust and support PrEP adherence.

A conceptual pathway for developing a women-centred PrEP programme. Foundational domains at the base include understanding lived realities, including ‘seasons of risk’, interpersonal trust, and intimate partner violence. These foundations inform the identification of women’s concerns and barriers to PrEP engagement, which in turn guide the design of services around women’s preferred access points and utilizing the community’s potential impact on adherence. Building on these layers, public health messaging and promotion of PrEP strengthen reach and demand, culminating in a PrEP program that may lead to improved uptake and adherence.

## 12. Conclusions

Improving PrEP uptake and persistence among women requires a multifaceted approach that addresses both clinical and structural barriers. The findings presented in this article underscore the need for tailored, women-centred interventions that move beyond a ‘one-size-fits-all’ approach [52,144], especially in the context of vast regional practices and among various communities. Understanding the concerns of women identified within the literature offers valuable insights for women-centred PrEP programmes. While this review did not include the discussion of long-acting PrEP, women report injectable PrEP as highly desirable [55,62], highlighting the potential of long-acting PrEP to improve uptake and adherence. Social factors like IPV, interpersonal trust between women and their partners, community stigma, and peer influences will continue shaping adherence and uptake across PrEP delivery methods. By offering discreet HIV prevention options aligned with women’s preferences for choices, long-acting injectables may further strengthen women-centred PrEP programmes. This includes tailored public health PrEP messaging to counter myths and emphasize empowerment; leveraging community- and peer-based support to provide trusted information and recognize IPV and the complex relationship dynamics as factors that affect PrEP use. Ultimately, women-centred PrEP programmes hold the potential to close the global gender-based gap in HIV prevention and advance equity while addressing persistent barriers. Policy-level interventions such as policy reform, national PrEP strategies, and sustained financial investment are critical to support equitable PrEP uptake among women [18]. Decriminalizing sex work and substance use could create safer healthcare environments that could lead to an uptake in PrEP and a reduction in HIV incidence [18]. However, their success will rely on context-specific implementation across regions that focus on the lived realities of women, considering variations in population demographics, sociocultural norms, healthcare infrastructure, and resource availability; all of which influence women’s environment and access to HIV prevention services.

## Data Availability

The data that support the findings for this manuscript are available from the corresponding author upon reasonable request.

## Abbreviations

The following abbreviations are used in this manuscript:

IPV	Intimate partner violence
TW	Trans women
AGYW	Adolescent girls and young women
SW	Sex workers
WWUD	Women who use drugs
NGO	Non-government organization
WHO	World Health Organization
